# Overview of US public health laboratory leadership development programmes and implementation opportunities

**DOI:** 10.1099/jmm.0.002032

**Published:** 2025-09-03

**Authors:** Emily L. McCutchen, Kathleen A. Street, Amanda M. Harvey

**Affiliations:** 1 Nebraska Public Health Laboratory, Omaha, NE, USA; 2 Association of Public Health Laboratories, Bethesda, MD, USA; 3 Agricultural Research Service, USDA, Manhattan, KS, USA

**Keywords:** Association of Public Health Laboratories, Centers for Disease Control and Prevention, development, director, laboratory, leadership, management, public health

## Abstract

**Introduction.** The public health laboratory system in the United States (USA) is extensive and focuses on the health of the nation’s population by providing testing for the identification and spread mitigation of public health concerns. Testing for communicable diseases, antimicrobial resistance, per- and polyfluoroalkyl substances, and much more is performed on human, animal and environmental specimens by federal, state and local level public health laboratories.

**Gap Statement.** Unique challenges exist for public health laboratory professionals, and individuals in this field are commonly appointed to leadership positions without any formal leadership or management training.

**Aim.** The authors aim to identify potential challenges for public health laboratory leaders and suggest how leaders may successfully navigate them. Additionally, this article aims to expand awareness of federally funded public health laboratory leadership programmes in the United States and when they may be applicable for prospective or current leaders.

**Methodology.** The authors reviewed publications and presentations to determine successful and unsuccessful leadership attributes in a public health laboratory context. They assessed federally funded leadership programmes in the USA via publications and publicly available information and interviewed select individuals who participated in or assisted in the development of these programmes.

**Results.** Six common challenges for public health laboratory leaders were identified and potential solutions are suggested. The Centers for Disease Control and Prevention (CDC) and the Association of Public Health Laboratories (APHL) provide four unique laboratory leadership training programmes for laboratorians interested in or currently employed within public health laboratories.

**Conclusion.** While the content of the federally funded programmes is different, they all aim to provide current and future public health laboratory leaders with the skills that they need to effectively guide and support laboratory staff through the critical and intense situations that public health laboratories commonly face.

**Impact Statement**

This article aims to expand awareness of the current landscape and challenges experienced in public health laboratory leadership development. Authors differentiate successful and unsuccessful leadership attributes and actions taken by laboratory leaders while sharing practices that may benefit readers’ implementation in their own laboratories. Furthermore, the article reviews available laboratory leadership development programs offered by the Centers for Disease Control and Prevention and the Association of Public Health Laboratories and the overall impact in recent US public health response.

## Data Summary

The authors confirm that all supporting data, code and protocols have been provided within the article or through supplementary data files.

## Introduction

Scientists seek education to obtain the knowledge and skills required for them to achieve specific roles. Given the value of education in the scientific community, it is common for scientists to possess multiple collegiate degrees, specialties and, in some instances, certifications, which require years of study to achieve. However, consider this: When was the last time that you met a laboratory leader who previously intended to hold a leadership role? If you can recall an instance, did they seek education or professional development training in advance of seeking a leadership role? How frequently do you see scientific doctoral degree holders concurrently possess formal education in leadership, such as a Master of Business Administration (MBA) degree? You may not be able to recall individuals who embody the intention of these questions. If so, why may that be the case?

## Public health laboratories in the USA

Due to the vast and varied landscape of the USA, the public health laboratory system is extensive and is comprised of federal, state and local level public health laboratories providing environmental, agricultural and human clinical testing. These laboratories, which are unique and separate from clinical laboratories, are located in every state and territory, including the District of Columbia, and provide testing services for the defined area(s) that they serve. The testing performed by public health laboratories is primarily focused on the population, as opposed to acute care, which distinguishes them from clinical laboratories. Among many other things, the testing performed by public health laboratories ensures that water is safer to drink, food is safer to eat, newborns are screened for genetic disorders, antimicrobial resistance is monitored and infectious disease outbreaks are identified and controlled. Additionally, these laboratories maintain a highly qualified workforce prepared and trained to respond to biological and chemical terrorism events. This work is achieved through multiple collaborations with national entities, such as the Centers for Disease Control and Prevention (CDC), the Environmental Protection Agency and the Food and Drug Administration, as well as global organizations with matching goals, such as the World Health Organization (WHO) [1,2].

In the USA, every citizen’s life is affected daily by work performed by public health laboratories. However, the coronavirus disease 2019 (COVID-19) pandemic has placed intense additional stress on public health laboratorians. According to the Public Health Workforce Interests and Needs Survey conducted in 2021, approximately one-third of the US public health workforce workers were considering leaving their jobs within a year for reasons other than retirement, and more than half of the public health workforce workers reported at least one symptom of post-traumatic stress disorder [3]. A follow-up survey in 2024 revealed that 29% of US public health workers aged 35 and under, who make up 25% of the total US public health workforce, intend to leave their position in the next year [4]. Position vacancies and the loss of institutional knowledge will affect public health laboratory leaders’ ability to perform their job duties effectively, and managers may find it more difficult to relinquish daily tasks in the laboratory to focus on managerial roles and leadership development. Laboratory leadership development programmes offered to US public health laboratorians assist in the development of strong formal and informal laboratory leadership and support the effort to build and retain current staff while recruiting and building future public health laboratory leaders.

## What is laboratory leadership?

A leader is defined as an entity responsible for ‘directing, commanding, or [serving as a] guiding head, [such] as of a group or activity’ [5]. While the definition implies that a leader must have followers, it does not include any specific attributes or rank that a leader must possess. However, the business world often conflates leadership with management, although many individuals lead others in the workplace without official recognition, and many managers fail to lead their staff effectively. Others believe that leadership, and thus leaders, are defined in terms of tasks, rather than a role or position. For example, the Center for Creative Leadership ‘[believes] Leaders are people, who in connection with others, accomplish [tasks]…’ [6]. Furthering the focus on tasks, Rowe indicates that common attributes of effective everyday leaders include focusing on the success of others and oneself by helping others succeed, ensuring appropriate recognition at all levels and engaging in internal reflection for self-awareness and refining one’s skills and competencies [7].

Due to the complexities in definition, leadership is commonly categorized into two groups: formal and informal. Recognition of both the formal and informal forms of leadership is essential to fully understanding leadership aptitude and development. Formal leaders are selected for or elected to positions where they are given the power to make decisions on behalf of others and often direct them toward achieving goals. These positions usually come with official titles to designate the individual as an authority. The titles and authority make this form of leadership more recognizable. On the other hand, informal leaders are trusted and respected within teams. They influence and guide their peers without an official designation of authority. Identifying such individuals, encouraging their growth, and including them in conversations about goals and organizational changes can make formal leaders more successful [8].

The necessity of supporting informal leaders is particularly true in the laboratory setting, where formal leaders need the support of informal leaders to solve complex, high-consequence problems requiring a multi-disciplinary approach while incorporating the needs of various collaborators. To function properly, a laboratory must balance the need to respond quickly to emergencies and evolving healthcare needs while ensuring that daily testing proceeds quickly and accurately. An informal leader, such as a laboratorian, may not be a laboratory director or a virology supervisor, yet they may support formal leadership by overseeing method validations, setting the vision and activities of quality improvement projects or serving as a senior chemist where they foster scientific expertise while assuring the competency of newer laboratory staff. This example of informal leadership allows formal leaders to gain more time and energy to focus on high-level decisions and objectives. Successful informal and formal leader relationships can create a strong base for achieving ongoing success while effectively responding to public health emergencies. Recognizing that both forms of leadership are equally important and encouraging the development of both groups is imperative to an effective and functioning laboratory.

## Current laboratory leadership constraints

Current laboratory leaders understand it is common for laboratorians to be promoted into leadership positions because they have specialized technical knowledge and skills, having demonstrated them well in projects and prized laboratory activities. In other words, new laboratory leaders are often excellent ‘doers’ and ‘knowers’ who fall into leadership instead of deliberately cultivating essential leadership skills. They need to evolve into leadership and change practices that may have made them successful in other roles.

As a result, common leadership pitfalls (and solutions) in laboratorians include the following:

1. Leading the team while the leader continues to execute tasks. Instead, effective leaders grow a team’s skills, so others (instead of the leader) become excellent ‘doers’ [7].

2. Communicating needed actions by instructing others how to perform a task, which can be perceived as micromanagement. Instead, effective leaders convey what needs to occur or the desired outcome, which enables others to determine their journey towards task completion (and learn skills and thought processes along the way) [7,9].

3. Becoming overcommitted and overscheduled:

- This can be a byproduct of a laboratory leader not letting go of their previous responsibilities, instead continuing to perform them in a leadership role. As with the previous points, this too can be mitigated by effectively transitioning tasks to team members. Furthermore, strong resource management and boundaries can improve this situation [9]. Sometimes trade-off conversations need to be held with other leaders, putting resource constraints into perspective with an ‘if this…then that…’ demonstration of logic. For example, an effective laboratory leader may express this as, ‘If we release test results the same day, then the team must work over-time which is an increased cost and will lead to burn-out’.
- Deliberate planning of priorities and activities can also ensure that leaders do not overpromise and underdeliver [9]. While not everything can be planned in a VUCA (volatile, uncertain, complex and ambiguous) world, the recent pandemic has shown that the unexpected can be difficult to predict [10], especially in a laboratory that responds to emerging infectious diseases. According to Udemy Business, leaders should plan to use 65–80% of their and team members’ total time, saving the remainder for unknown priorities that always arise [9]. Effective laboratory leaders recognize it’s easier to negotiate commitments up front than to experience stress, burnout, morale loss and disappointment from the unexpected happening. Effective leaders expect and plan for VUCA.

4. Thoroughly conveying details at a level that is not right for their audience [7]. For example, an ineffective leader may describe newborn screening to the public in terms of genetic tests and amino acids. Conversely, an effective leader may liken the same essential public health laboratory test to situations that the same audience can relate to, such as popular movies or well-known cases. This is because an effective leader knows that a basic understanding (even if not completely accurate) makes a larger impression than overly technical terminology.

5. Operating with intense laser focus, concentrating on details to the extent that the bigger picture is not seen or considered. Laboratory leaders at all levels are responsible for special outputs that often connect with a laboratory’s mission and vision. When a leader can articulate the bigger picture for a team’s task (and connect everyone’s successes to the big picture), it centralizes them around a common purpose, which encourages staff motivation and retention [9].

6. Adopting professional loneliness due to a lack of connections and networking with others [9]. In a laboratory, it is easy to operate within the siloes of workflows and scientific specialties. Effective leaders mitigate this by building a network of peers and a community of practice.

## US leadership development programmes

In the USA, there are currently four federally funded public health laboratory leadership development programmes: Laboratory Leadership Service (LLS), Emerging Leader Program (ELP), Laboratory Director Board Examination Boot Camp and the Laboratory Leaders of Today (LLOT). These leadership development programmes offer fully immersive and part-time opportunities for both formal and informal laboratory leaders at various stages in their careers. The LLS is fully supported by the CDC, whereas the remaining three programmes are supported by a cooperative agreement between the CDC and the Association of Public Health Laboratories (APHL) under the parent programme, Career Pathways. The collaborative efforts between the CDC and the APHL for laboratory leadership development can also be noted in their involvement as partner organizations for the development of the WHO’s Global Laboratory Leadership Programme, discussed below [11].

## Laboratory leadership service

The CDC’s LLS is unique in that it requires 2 years of full-time participation and likely relocation, hindering the programme participants’ ability to retain previous full-time employment. During the 2 years, the LLS programme allows doctoral applicants the ability to become fully immersed in public health laboratory activities, regardless of previous public health experience [12].

The LLS focuses on the development of skills necessary to be successful public health laboratory leaders and was initially an in-agency programme modelled after the CDC’s Epidemic Intelligence Service, a fellowship programme focused on applied epidemiology. Though participants have differing backgrounds, the two programmes participate in side-by-side training, encouraging collaboration and a deep understanding of the critical epidemiology-laboratory relationship [13,14]. At conception, the LLS programme heavily emphasized laboratory quality and safety, in addition to other training topics such as bioinformatics, advanced communication and laboratory programme management. The programme is highly competitive, with only 6–8 applicants accepted annually, accounting for ~16% of all eligible applicants. Recent class sizes have increased but acceptance remains highly competitive [13]. Following a 1-month orientation in Atlanta, Georgia, accepted fellows are placed at the CDC or at local or state public health laboratories where they focus on completion of ten core activity learnings (CALs). These CALs are based upon competencies of laboratory professionals established by the CDC and APHL and provide hands-on opportunities to work beside mentors and supervisors to participate in and perform activities aimed at developing skills necessary to lead public health laboratories [14]. In addition to the completion of the CALs, the programme includes applied and experiential training exercises, professional development training, workshops and an opportunity for each fellow to participate in a public health response [14].

Former LLS fellow, Dr. Matthew Martin, stated, ‘There are three main areas that I see that LLS has been beneficial to my leadership development. First, it has provided structured and scientific opportunities to evaluate my leadership strengths and weaknesses. Second, it has provided opportunities for me to practice in areas where my leadership skills aren’t as strong and to improve in those areas. Third, it has provided me with the opportunity to hear from and speak with a variety of people in leadership positions in public health and to learn about their career and leadership development journeys’ [15].

## Emerging leader program

The APHL works globally and within the USA to strengthen laboratory systems by serving the public’s health while partnering with external entities, such as US federal governmental offices and state and local governmental laboratories [16]. In addition to providing guidance regarding public health policies, fostering laboratory innovations and providing laboratory guidance, APHL prioritizes public health laboratory workforce development to promote the development of existing and future laboratory leaders [17].

One such APHL effort is the ELP, which consists of an annual hybrid virtual and in-person programme for current public health laboratory professionals on all levels of the leadership spectrum. To increase programme accessibility, APHL has recently added a fully virtual programme option for upcoming cohorts. Unlike LLS, the ELP does not require full-time participation; thus, participants maintain their employment at their home public health laboratory during programme participation. The ELP is for any public health laboratorian who exhibits leadership potential regardless of their current job level classification within the laboratory. Not all ELP participants supervise others, and true to everyday leadership, not all leaders aspire to become supervisors. Since its inception nearly 20 years ago, the ELP has enabled over 230 participants from 19 cohorts of state, local, environmental, agricultural, federal and international (Canadian) laboratories to engage in skill development workshops, undergo networking opportunities and develop/apply core leadership skills through exercises [18]. Skills in teamwork, clear communication, collaboration, relationship building and networking are learnt through a variety of exercises. In addition, each annual cohort must work together to develop a large project that responds to public health laboratory challenges, such as toolkits, videos and trainings to enhance essential laboratory leadership skills or increase awareness of the importance of public health laboratories [19]. This highly successful programme has resulted in more than 30% of ELP graduates currently serving as senior laboratory leaders (laboratory directors or assistant/deputy laboratory directors) and an additional two graduates completing the University of South Florida’s doctorate in public health (DrPH) programme, another APHL–CDC initiative [20].

Upon graduation, participants join a community of alumni, the Emerging Leader Alumni Network (ELAN), which serves as a continuation of the programme’s momentum and a way for alumni to participate in future leader development. Alumni give back to the current ELP cohort by reviewing applications for programme admission, serving as individual coaches to the current cohort of participants, assisting with and encouraging networking and serving as a sounding board for the current ELP cohort’s project and other workforce-related needs.

As with all programmes, APHL’s ELP has evolved to meet current public health laboratory needs; however, through these transitions, key goals have remained the same. Dr. Peter Iwen, D(ABMM), Laboratory Director of the Nebraska Public Health Laboratory and participant in the inaugural ELP Cohort 1, stated that the single most impactful thing learnt during his time in ELP was ‘power of collaboration with peers’ [21]. Due to Dr. Iwen’s successful experience, he has since nominated multiple individuals within the laboratory to ensure the long-term success of the Nebraska Public Health Laboratory. Dr. Iwen believes ‘participation in leadership training in a group setting empowers individuals to better understand their style as a leader and provides guidance that can be brought back to the laboratory, resulting in a more successful laboratory environment’ [21].

Prior to joining APHL as the Chief Learning Officer, Dr. Christine Bean also saw the direct benefits associated with nominating her laboratory staff for participation in ELP: ‘During my tenure as Laboratory Director at the New Hampshire Public Health Laboratories (2005–2021), I nominated six laboratory supervisors to participate in ELP. I saw future leaders in my staff. The ELP was key to their acknowledgement of their interest in leadership development, which gave them the confidence and training to take on new roles. I saw each of these laboratorians take the next steps in their career pathways after completing the ELP. Examples include one who became the Laboratory Information Management System (LIMS) Administrator, one who moved from supervisor to Bureau Chief, and another who is now in a manager position at APHL. I saw the value of ELP and was grateful that APHL hosted such an excellent programme for members’ [22].

## The laboratory director board examination boot camp

In the USA, laboratory personnel requirements are dictated by the Clinical Laboratory Improvement Amendments programme, which was approved by Congress in 1988 to ensure the production of high-quality human clinical laboratory testing results. Per title 42, part 493 subpart M, to be eligible to serve as a high complexity laboratory director in the USA, an individual must, at a minimum, be a licensed Doctor of Medicine or Osteopathy with either certification in anatomic or clinical pathology or medical residence laboratory training and supervisory experience or a science-based doctoral degree with US Department of Health and Human Services (HHS)-approved board certification [23]. While these advanced degrees and certifications are associated with the higher levels of leadership within laboratories, they do not automatically indicate that the individuals in these positions have received substantial training in areas thought to be key to leader success, such as communication, effective planning and self-reflection.

APHL’s Laboratory Director Board Examination Boot Camp is a multi-week virtual programme designed to help jump-start the studying process for the board certification exam that is required to direct high-complexity laboratory testing in the USA should the individual hold a science-based doctoral degree. The programme’s goal is to increase the number of laboratorians pursuing and passing board certification exams and, thus, become eligible to serve as high-complexity laboratory directors. While previously described leadership development programmes focus on teaching skills such as communication and teamwork, the Laboratory Director Board Examination Boot Camp focuses on preparing future laboratory directors for their board examinations. During weekly ‘boot camp’ sessions, a panel of board-certified laboratory directors and other APHL volunteers provides advice and guidance regarding the board exam and its topics, while suggesting additional resources related to leadership positions and board certification requirements. The facilitators administer a Kahoot! Online game format for participants. This allows for participant anonymity and data collection while utilizing an engaging and competitive environment to learn and retain information. Each session focuses on a different topic, and between sessions, future and past exam takers are given a forum to connect and share knowledge and study ideas. To further aid in success, participants are also provided access to Quizlet, a site that houses board examination study resources, including over 6,000 online flashcards.

The programme does not limit participation and accepts all individuals who are interested in taking a board certification exam in pursuit of director-level positions within public health laboratories. APHL has learnt that participation varies depending on the topic, but in Spring 2023, near-weekly sessions ranged from 30 to 50 participants [18,20].

## Laboratory leaders of today

While the ELP is for any public health laboratorian and the Laboratory Director Board Examination Boot Camp is for those seeking board certification to become laboratory directors, the LLOT is a 18-month hybrid virtual and in-person programme for new laboratory directors, and assistant or deputy directors from state, local, environmental and agricultural laboratories who have been in their role for 1 year or less. Recently, APHL expanded the programme audience to include senior laboratory leaders who have a career trajectory of potentially rising to a laboratory director role. According to APHL, 40% of state/local laboratory directors are new to their role [20]. This programme addresses critical operational issues in member laboratories while fostering networking and the development/application of core leadership skills. Key programme attributes include extensive participant collaboration and discussions. Sessions are supported by external partners, the APHL Board of Directors, and established laboratory directors and assistant/deputy lab directors [20].

LLOT was formerly known as the National Lab Director Orientation (NLDO). In 2022, Dr. Bean transformed NLDO into LLOT because, ‘I participated in the NLDO in 2005 and knew the value of being oriented to a new role and joining a cohort of my peers for networking and collaboration. When I started as [APHL’s Chief Learning Officer] in 2021, the NLDO had not been offered since before the pandemic, and I saw an opportunity to evolve the programme into something more. Many laboratory directors had retired, and the new group of leaders was invited to a year-long programme for training, discussions and sharing best practices. [APHL] hosted 68 new directors/deputies during the first cohort of LLOT’ [22].

In September 2023, LLOT Cohort 1 concluded, and participants transitioned to the alumni group (Laboratory Leaders of Today Alumni/LLOTA), which operates similarly to ELAN. In October 2024, 14 participants graduated from Cohort 2. Participants interact through an electronic discussion board, and volunteers from Cohort 1 joined a selection of Cohort 2 participants on a planning committee for the current cohort. Furthermore, the current LLOT cohort and its alumni have access to potential cross-development opportunities offered by APHL, in collaboration with the CDC [18].

The leadership development programmes offered by the APHL, such as the Board Examination Boot Camp and LLOT, are designed to assist public health laboratorians at various places in their career path, should they choose to advance to laboratory director. In instances where a candidate could qualify for both ELP and LLOT programmes, a decision is made as to which programme is more appropriate for them to undergo. Given the ELP and LLOT’s current time commitments, it is not feasible for a candidate to undergo both programmes at the same time. As Dr. Bean notes, although concurrent participation in the programmes is not recommended, there is overlap in participants between both programmes: ‘Many of the new laboratory directors/deputies participated in the ELP, which demonstrates the value of the programme to develop future leaders in public health laboratory science’ [22].

To encourage lasting support, ELP, LLOT and the Board Examination Boot Camp all utilize communities of practice cultivated by APHL through an electronic platform called ColLABorate [24]. This platform, which is used in many APHL programmes and not just leadership development, allows programme graduates of all cohorts to participate in ongoing electronic communication to share resources, best practices, post-inquiries and participate in discussion threads with peers and mentors [23]. The ability to have instant communication with individuals who have, or currently are, in your same situation allows for further and sustained networking and collaboration opportunities while building a sense of community for public health laboratory leaders.

## The importance of laboratory leadership development

The laboratory leadership development programmes provided by the CDC and APHL are crucial to the ongoing success of public health laboratories in the USA. The COVID-19 pandemic stressed public health laboratories, as evidenced by the findings in the 2021 Public Health Workforce Interests and Needs Survey. As previously mentioned, nearly one-third of state and local public health laboratory professionals indicated that they were considering leaving their current organization within a year’s time [3]. Further analysis of separation trends indicates that up to half of the governmental public health workforce could leave by 2025 which would leave the US public health system crippled for future outbreak or pandemic response [25]. However, the survey also indicates that the top reasons individuals ages 35 and younger in 2017 stay within the organization are supervisor satisfaction, job satisfaction and pride in the organization and its mission [25]. These organizational attributes all fall under the mission of laboratory leaders and further highlight the need for sustained and robust laboratory leadership development.

Appropriate laboratory leadership development has a positive impact on US public health. One such example of this is the communication-based skills application provided as part of ELP training, which trains leaders on how to tailor communication based on audience needs. For instance, when providing laboratory tours to the media, legislators and other interested parties, laboratory leaders may choose to relate laboratory tests to popular movies and books so that tour goers can better understand the laboratory’s function and purpose and advocate for the laboratory’s financial needs and public health purpose [18]. The same skills can be applied to improve internal communication and mitigate bureaucracy. For laboratories that are part of a larger organizational structure (such as a state public health laboratory), providing laboratory tours to administrative areas that support laboratory functions can educate organizational partners and improve the turnaround time of organizational processes. This occurred recently when a public health laboratory provided a tour for employees serving in areas the lab is dependent upon (legal, procurement and contracts). The laboratory employees reviewed specific processes and documents with protracted reviews and scheduled tour stops and prepared descriptions of how not being able to purchase specific laboratory supplies, reagents and instrumentation would negatively impact the laboratory’s operations and public health. Processes moved swiftly after the information was shared [18].

The leadership development programmes described also provide participants with networking opportunities beyond state/county lines and organizational silos, and the relationships forged from these opportunities can provide lasting public health impact. For example, due to a natural disaster, one state laboratory engaged in testing services from a different state laboratory. Although the testing relationship was in effect for multiple years, the laboratory performing testing was not receiving compensation from the submitting laboratory. One leader from each laboratory participated in the same cohort of the ELP and formed a working relationship. Together, they identified the non-payment issue as a miscommunication and resolved the issue. When public health services have no payor, they pose a burden upon the public. The formation of working relationships between laboratory leaders allowed for the testing relationship to continue [18].

Some ELP participants return to their laboratories and form home-grown leadership programmes. To ensure the success and robustness of development, programmes should have laboratory director support and a central leader who can develop or fund the outsourcing of leadership development content. However, some laboratories may find benefit in leadership programmes that do not share content but instead allow for routine meetings with other aspiring and developing leaders to share challenges and best practices. Either format can increase a leader’s confidence and broaden their working relationships with others, enabling them to collaborate to solve organizational problems and retain their workforce by being a model leader to work for [18].

## Other leadership solutions

Recognizing the need for standardized laboratory leadership on a global scale the Food and Agriculture Organization of the United Nations, the WHO, the World Organisation for Animal Health, the European Centre for Disease Prevention and Control, the CDC and the APHL developed the Laboratory Leadership Competency Framework which details nine essential competencies and provides the foundation for the Global Laboratory Leadership Programme (GLLP) [11,26]. The GLLP is a comprehensive, competency-based One Health leadership initiative designed to foster and mentor current and emerging laboratory leaders to build, strengthen and sustain national laboratory systems across human, animal and environmental health sectors. The GLLP promotes country ownership and sustainability, engaging countries to adapt the program to county-specific needs and incorporate the programme into their larger workforce development efforts [11].

If it is not feasible to implement a laboratory leadership programme, alternate leadership solutions utilized in these formal programmes, such as coaching and mentoring, can be implemented. Though commonly hard to quantify, multiple studies have found statistical evidence supporting the benefits of mentoring in a laboratory setting [27,29]. Due to intangible metrics, quantification of mentorship is difficult, but many studies are quantifying the development or optimization of a quality management system to determine the positive mentoring impact. Additionally, the positive impact of mentoring in the sciences is so well recognized that the high-impact scientific journal *Nature* has an annual Nature Awards for Mentoring in Science, which recognizes lifetime and mid-career scientists ‘who have made outstanding contributions to the mentoring of early-career scientists’ [30,31].

## Abbreviations

APHL: Association of Public Health Laboratories
CALs: core activity learnings
CDC: Centers for Disease Control and Prevention
COVID-19: coronavirus disease 2019
ELAN: Emerging Leader Alumni Network
ELP: Emerging Leader Program
GLLP: Global Laboratory Leadership Programme
LLOT: Laboratory Leaders of Today
LLS: Laboratory Leadership Service
NLDO: National Lab Director Orientation
VUCA: volatile, uncertain, complex and ambiguous
WHO: World Health Organization
